# Exploring gut microbiota alterations in Parkinson’s disease: insights from a 16S amplicon sequencing Eastern European pilot study

**DOI:** 10.3389/fnins.2025.1654995

**Published:** 2025-10-31

**Authors:** Ovidiu-Dumitru Ilie, Irina-Cezara Văcărean-Trandafir, Roxana-Maria Amărandi, Ilinca-Bianca Nita, Petru-Romeo Dobrin, Mara Doroftei, Iuliu-Cristian Ivanov, Gheorghe Savuta, Boris Kirov, Bogdan Doroftei

**Affiliations:** ^1^Regional Center of Advanced Research for Emerging Diseases, Zoonoses and Food Safety, Department of Public Health, Faculty of Veterinary Medicine, “Ion Ionescu de la Brad” Iasi University of Life Sciences, Iasi, Romania; ^2^TRANSCEND Research Centre, Regional Institute of Oncology, Iasi, Romania; ^3^Technical University of Sofia, Sofia, Bulgaria; ^4^Department of Psychiatry, Faculty of Medicine, “Grigore T. Popa” University of Medicine and Pharmacy, Iasi, Romania; ^5^“Socola” Institute of Psychiatry, Iasi, Romania; ^6^Faculty of Medicine, “Grigore T. Popa” University of Medicine and Pharmacy, Iasi, Romania; ^7^Faculty of Automatics, Technical University of Sofia, Sofia, Bulgaria; ^8^BioInfoTech Lab, Sofia Tech Park, Sofia, Bulgaria; ^9^Department of Mother and Child, Faculty of Medicine, “Grigore T. Popa” University of Medicine and Pharmacy, Iasi, Romania; ^10^“Cuza Voda” Clinical Hospital of Obstetrics and Gynecology, Iasi, Romania; ^11^Origyn Fertility Center, Iasi, Romania

**Keywords:** Parkinson’s disease, gut microbiome, dopaminergic agonists, amplicon sequencing, MiSeq, 16S rRNA

## Abstract

**Introduction:**

Parkinson’s disease (PD) is a neurodegenerative disorder increasingly associated with alterations in gut microbiota through the gut–brain axis (GBA). Despite growing global interest, studies examining microbiota composition in Eastern European populations remain limited.

**Methods:**

We profiled the gut microbiota of 59 Romanian individuals using 16S rRNA gene sequencing targeting the V3–V4 region. After quality filtering, 39 subjects (19 PD patients and 20 healthy controls [HC]) were retained for downstream analysis. Clinical metadata were collected to assess potential confounders, including age, sex, metabolic parameters, lifestyle, and comorbidities.

**Results:**

PD patients differed significantly from HCs in glycemia (*p* = 0.02), cholesterol (*p* = 0.027), and LDL levels (*p* = 0.047), and more frequently presented with restrictive diets and comorbidities such as cardiovascular disease and diabetes. While α-diversity metrics did not differ significantly between groups, principal coordinate analysis (PCoA) based on Aitchison distance showed moderate compositional separation. Permutational multivariate analysis of variance (PERMANOVA) confirmed that disease status was a significant driver of gut microbiota composition (*R*^2^ = 5.3%, *p* = 0.002), independent of clinical and lifestyle covariates. Sparse partial least square linear discriminant (sPLS-DA) identified several genera distinguishing PD from HC, with *Mogibacterium* and *RikenellaceaeRC9* gut group enriched in PD, and several known short-chain fatty acid (SCFA)-producing genera (*Fusicatenibacter*, *Lachnospiraceae UCG-001*, *Butyricicoccus*, *Anaerostipes*) enriched in HCs. Linear discriminant analysis (LDA) Effect Size (LEfSe) corroborated these findings, confirming the differential abundance of several SCFA-producing genera in the HC group.

**Discussion:**

Our results reveal distinct microbial signatures associated with PD in this Romanian cohort, marked by a consistent depletion of SCFA-producing bacteria in patients. These findings support the role of gut microbiota in PD pathophysiology and underscore the need for further studies in Eastern European populations.

## 1 Introduction

PD is the second most common neurodegenerative disorder after Alzheimer’s disease (AD) (Nichols et al., 2022), affecting 2–3% of individuals aged 65 and older (Poewe et al., 2017; Shrimanker et al., 2024). Its global prevalence is expected to rise from 11.8 to 25.2 million by 2050, a 112% increase (Dorsey et al., 2018; Ben-Shlomo et al., 2024; Su et al., 2025). As reported in the Global Burden of Disease Study (GBD) 2021, PD accounted for approximately 7.47 million disability-adjusted life years (DALYs), reflecting a 161.8% increase since 1990, primarily due to population aging and the disease’s progressive course (Lampropoulos et al., 2022). PD also presents a gender disparity, with a male-to-female incidence ratio of 1.5:1 (Elbaz et al., 2016), and a greater burden in men (117.47 DALYs per 100,000) than in women (68.56) (Luo et al., 2025; Peng et al., 2025).

The pathological hallmark of PD is the degeneration of dopaminergic neurons in the substantia nigra pars compacta (SNpc), leading to dopamine deficiency and classical motor symptoms such as bradykinesia, resting tremor, rigidity, and postural instability (Dauer and Przedborski, 2003; Moustafa et al., 2016). Increasingly, PD is recognized as a multisystem disorder with widespread α-synuclein (α-syn) pathology beyond the nigrostriatal pathway, affecting regions like the locus coeruleus and cerebral cortex (Braak et al., 2003b; Surmeier et al., 2017). This explains its broad range of non-motor symptoms, including cognitive decline, mood disorders, autonomic dysfunction, and hyposmia, than can precede motor features by a decade, significantly influencing the quality of life (QoL) (Chaudhuri et al., 2006; Poewe, 2008; Leite Silva et al., 2023).

Despite advances in symptom management, particularly for motor fluctuations, early diagnosis remains challenging, and no therapies currently halt disease progression (Rees et al., 2018; Fabbri et al., 2023). PD pathogenesis is considered multifactorial, involving mitochondrial dysfunction, oxidative stress (OS), and neuroinflammation (Pajares et al., 2020; Dorszewska et al., 2021; Henrich et al., 2023). The dual-hit and Braak hypotheses suggest that PD may originate in peripheral sites like the olfactory bulb or enteric nervous system (ENS), potentially triggered by environmental agents that induce α-syn misfolding (Braak et al., 2003a; Hawkes et al., 2007, 2009). Misfolded α-syn may propagate in a prion-like manner via the vagus nerve, reaching the midbrain and contributing to Lewy body formation (Braak et al., 2002; Dickson et al., 2009; Hawkes et al., 2010; Cryan and Dinan, 2012; Cersosimo, 2015). α-syn aggregates in the ENS, including the Meissner and Auerbach plexuses, possibly representing an early-stage pathology (Cersosimo, 2015). Moreover, emerging evidence suggests that gut microbiota dysbiosis may influence neuroinflammatory processes and contribute to PD progression (Cryan and Dinan, 2012).

The GBA has thus gained attention in PD research. The gastrointestinal (GI) tract may act as early sensor and driver of neurodegeneration, with the gut microbiota playing a key role in immune modulation, barrier integrity, and neurochemical production (Sun X. et al., 2022). Dysbiosis in PD is characterized by reduced SCFA-producing bacteria (e.g., *Faecalibacterium* and *Roseburia*) and increased *Akkermansia* (Nishiwaki et al., 2020b). This imbalance may impair SCFA production, especially butyrate, compromising the intestinal barrier and enabling translocation of pro-inflammatory molecules like lipopolysaccharide (LPS) (Dalile et al., 2019; Silva et al., 2020; Wachamo and Gaultier, 2025).

Recent studies from various regions highlight geographical differences in gut microbiota composition among PD patients. A Polish cohort study reported an increased abundance of *Akkermansia muciniphila* (phylum Verrucomicrobiota), *Parabacteroides merdae* (phylum Bacteroidota), and *Holdemanella biformis*, formerly *Eubacterium biforme* belonging to the Bacillota phylum (previously Firmicutes) (Zapała et al., 2021). These taxa are associated with mucin degradation and have been implicated in increased gut permeability, potentially contributing to systemic inflammation (Zapała et al., 2021). In contrast, a Russian study observed a reduction in *Prevotella*, a genus known for its anti-inflammatory properties and its role in maintaining mucosal integrity (Petrov et al., 2017). Meta-analyses consistently reveal significant alterations in gut microbiota composition in PD patients compared to HCs, marked by a reduction in anti-inflammatory, SCFA-producing genera such as *Faecalibacterium* and *Prevotella*, alongside an enrichment of potentially pro-inflammatory taxa, including *Akkermansia* and members of the *Enterobacteriaceae* family (Nishiwaki et al., 2020b; Romano et al., 2021; Shen et al., 2021; Toh et al., 2022; Bai et al., 2024).

However, microbiota metrics such as α-diversity remain inconsistent as some studies report increased diversity due to rare taxa expansion and reduced dominant species, while others find decreased richness and evenness (Romano et al., 2021; Bai et al., 2024). β-diversity, on the other hand, more consistently reveals altered microbial composition in PD versus controls, though variability exists across studies (Nishiwaki et al., 2020b; Romano et al., 2021; Toh et al., 2022; Bai et al., 2024). A notable limitation in the current literature is the underrepresentation of Eastern European populations, particularly from countries like Romania, where microbiome research remains scarce despite potential regional differences in diet, genetics, antibiotic exposure, and healthcare accessibility that may influence microbial composition and disease manifestation. As a genetically and nutritionally distinct Central-Eastern European population, Romanian cohorts offer valuable insights into how environmental and lifestyle factors modulate the GBA in PD. Most existing studies focus on cohorts from Western Europe, North America, and East Asia (Romano et al., 2021), leaving a gap in understanding how regional factors influence gut microbiota in PD. To address these inconsistencies, particularly in underrepresented regions, our pilot study aims to generate a preliminary microbial profile of Eastern European PD patients compared to HCs.

## 2 Materials and methods

### 2.1 Ethical approval

This study was conducted in accordance with the Declaration of Helsinki regarding Human Rights and other relevant National and European regulations governing Biomedical Research. The Ethics Committee of the “Socola” Institute of Psychiatry, Iasi, Romania, approved the protocol and informed consent (no. 29475, dated 25.09.2024), and all participants provided written informed consent prior to enrollment. While HCs joined voluntarily, PD patients were first evaluated by their psychiatrists and neurologists to ensure eligibility. Individuals deemed at risk due to health or mental status were excluded. The informed consent outlined the study’s purpose, procedures, potential risks and benefits, participant rights, including withdrawal at any time, confidentiality safeguards, compensation details, and how data would be used for scientific purposes.

### 2.2 Participants

A total of 59 Romanian individuals were enrolled in this study, including 26 males and 33 females. Of the initial 59 participants, five were excluded due to insufficient DNA concentration for sequencing. An additional 15 samples were removed after quality control filtering for sequencing depth (<5,000 reads). As a result, the final cohort comprised 39 individuals (20 HCs and 19 PD), 19 males and 20 females. Of these, 20 HCs (7 males and 13 females) were opportunistically recruited during routine laboratory visits. The remaining 19 subjects were PD patients (12 males and 7 females) diagnosed with PD, based on the International Classification of Diseases-10 (ICD-10) by psychiatrists and neurologists. PD patients were admitted between March and August 2024 from Bârnova and Șipote external units of the “Socola” Institute of Psychiatry, a tertiary-care center located in Iasi, Romania.

Clinical parameters such as fasting glycemia, total cholesterol, HDL-cholesterol, LDL-cholesterol, and triglycerides were obtained from recent medical records. Participation in questionnaires was voluntary. PD patients completed one or more validated tools to assess cognitive and psychological status, including the Mini-Mental State Examination (MMSE), Hamilton Anxiety Rating Scale (HAMA), Hamilton Depression Rating Scale (HAMD), Global Assessment of Functioning Scale (GAFS), Brief Psychiatric Rating Scale (BPRS), and the Reisberg Global Deterioration scale.

Additional metadata collected for each participant included activity level (e.g., active, sedentary), and smoking status. Comorbidities were grouped into four clinically relevant categories: cardiovascular diseases, hepatic disorders, obesity, and diabetes mellitus. Each category was encoded as a binary (presence/absence) variable and later used as a covariate in multivariate analyses to control for potential confounding effects. Diet type was also recorded for each participant and, in most cases, was prescribed in accordance with clinical comorbidities (e.g., low sodium diets for cardiovascular disease, hypocaloric or hypoglycemic diets for obesity, and hepatic diets for liver-related conditions). Due to this close relationship, diet type and comorbidity variables were not analyzed simultaneously in multivariate models to avoid collinearity.

### 2.3 Inclusion and exclusion criteria

To ensure consistency and minimize confounding factors, participants had to meet the following inclusion criteria: (1) age over 18, (2) a confirmed diagnosis of idiopathic PD, (3) voluntary agreement to participate, (4) ability to provide a stool sample, (5) capacity to provide informed consent, (6) a stable antiparkinsonian medication regimen for at least 1 month, (7) no use of vitamins, omega-3 supplements, prebiotics, or probiotics in the last 2 weeks, and (8) no antibiotic or immunosuppressive treatment within the last 3 months. Conversely, individuals who met any of the exclusion criteria were automatically removed: (1) a diagnosis of atypical parkinsonism; (2) current dependence on illicit substances, (3) a history or clinical evidence of inflammatory gastrointestinal, hematological or autoimmune diseases; and (4) adherence to specific diets known to influence gut microbiota composition (e.g., vegetarian, vegan, ketogenic low-fat diet, gluten-free diet, or intermittent fasting). HCs were recruited from the same local community and underwent thorough physical and laboratory assessments to rule out any medical or mental conditions. They met the same inclusion and exclusion criteria as the PD group, with the exception of a PD diagnosis.

### 2.4 Sample collection

To preserve genetic material integrity, fecal samples were collected in sterile plastic containers and stored at −20 °C within 15–30 min post-collection. This method is consistent with evidence showing that short-term storage up to 24 h or longer does not significantly alter microbial composition when maintained at appropriate temperatures, regardless of the use of preservative buffers (Cardona et al., 2012; Choo et al., 2015; Tedjo et al., 2015; Moossavi et al., 2019). Samples were subsequently transported to the laboratory on dry ice and stored at −80 °C until further analysis. To ensure sample reliability considering that DNA is generally less prone to degradation over time compared to RNA, all stool specimens were processed within 1 year, a timeframe shown to maintain microbial stability in terms of structure, composition, and diversity (Li X.-M. et al., 2023).

### 2.5 DNA extraction procedure

Fecal DNA was extracted in batches of 12 samples to minimize cross-contamination risk. Approximately 350 mg of stool was processed using the NucleoSpin^®^ Soil kit (Macherey-Nagel, Düren, Germany) following manufacturer instructions with specific optimizations. Buffer SL2 and Enhancer SX were used, and the protocol was adapted to enhance lysis efficiency across both Gram-positive and Gram-negative bacteria (Văcărean-Trandafir et al., 2023, 2024).

### 2.6 Enzymatic and mechanical lysis

Samples were incubated overnight at 37 °C with 800 μL of SL2 buffer and 40 μL of proteinase K (Thermo Fisher Scientific, Waltham, MA, United States) (20 mg/mL ~ 600 U/mL). This was followed by a 1-h incubation at 37 °C with 50 μL of lysozyme (Sigma-Aldrich Co., St. Louis, MO, United States) (10 mg/mL ~ 40 KU/mL) and 3 μL of lysostaphin (Sigma-Aldrich Co., St. Louis, MO, United States). Afterwards, the samples were thoroughly vortexed for several minutes.

Mechanical lysis was carried out with a FastPrep-24™ homogenizer (MP Biomedicals, Irvine, CA, United States) at 6 m/s for 80 s, using MN Bead Tubes type A. Lysates were centrifuged twice at 11,000 × *g* for 2 min, and 700 μL of supernatant was transferred to a NucleoSpin^®^ Inhibitor Removal column. DNA was eluted in 30 μL of preheated SE buffer (80 °C) and stored at −20 °C. Negative controls, consisting of the buffer without fecal material, were included in each batch (Văcărean-Trandafir et al., 2023, 2024).

### 2.7 Evaluation and analysis of extracted DNA

The concentration (A260) and purity (A260/230 and A260/280 ratios) were assessed using a NanoDrop spectrophotometer (Thermo Fisher Scientific, Waltham, MA, United States). DNA integrity and fragment size were confirmed via gel electrophoresis on a 2% agarose gel stained with ethidium bromide, run in 1x TAE buffer at 180 V (Văcărean-Trandafir et al., 2023, 2024).

### 2.8 16S V3-V4 rRNA sequencing

Bacterial community profiling was performed by amplifying the V3-V4 region of the 16S rRNA gene using Illumina-adapted primers and protocol (Illumina, 2013; Văcărean-Trandafir et al., 2023). Each 25 μL PCR reaction volume contained 12.5 μL of 2X KAPA HiFi HotStart Ready Mix (Roche Holding AG, Basel, Switzerland), 2.5 μL of microbial genomic DNA, and 5 μL each of forward and reverse primers (1 mM). The cycling program included initial denaturation (95 °C for 3 min), 25 cycles of 30 s at 95, 55, and 72 °C, and a final extension (72 °C for 5 min). Amplicons were purified with Agencourt AMPure XP magnetic beads (Beckman Coulter, Brea, CA, United States) using a BIOMEK^®^ FXP automated workstation (Beckman Coulter, Brea, CA, United States) (Văcărean-Trandafir et al., 2023, 2024).

A second PCR was performed using 5 μL of the purified amplicons to incorporate dual indexes and sequencing adapters using the Nextera XT Index Kit V2 set A (Illumina, San Diego, CA, United States). The reaction included 5 μL of each Nextera XT Index Primer (10 M), 25 μL of 2X KAPA HiFi HotStart Ready Mix, and 10 μL of nuclease-free water, totaling 50 μL. The cycling program included an initial activation/denaturation step (95 °C for 3 min), 8 cycles of 30 s each at 95, 55, and 72 °C, and a 5-min extension at 72 °C. The final sequencing library was again purified with AMPure XP magnetic beads. During the library preparation, DNase-free water was used as a negative control. The DNA concentration was measured using a Qubit 4 fluorometer and the Qubit 1X dsDNA High Sensitivity Assay Kit (Thermo Fisher Scientific, Waltham, MA, United States). Barcoded amplicon libraries were normalized and pooled to a final concentration of 4 nM. Sequencing of the pooled libraries was carried out on the Illumina MiSeq platform using a 12 pM concentration of the samples with a 20% Phix control spike-in, employing a paired-end (2 × 300 bp) sequencing kit to target the V3–V4 region of the 16S rRNA gene (Văcărean-Trandafir et al., 2023, 2024).

### 2.9 Bioinformatics and statistical analysis

#### 2.9.1 Sequence pre-processing and denoising

All analyses were performed in R (version 4.3.2). Sequence processing was performed following the DADA2 pipeline (Callahan et al., 2016), with minor modifications, similar to what was described in our previous work (Văcărean-Trandafir et al., 2023, 2024). After removing primers, forward/reverse sequences were trimmed to 255/210 bases, and sequences containing ambiguous bases were discarded. After merging paired reads, chimeras were detected and removed, and only merged sequences >350 bases were kept for downstream analyses. Taxonomy was assigned using the Ribosomal Database Project (RDP) Naive Bayesian classifier implemented in DADA2, employing the SILVA database release 138 (80% bootstrap confidence) (Quast et al., 2013; McLaren and Callahan, 2021). Amplicon sequence variants (ASVs) unclassified at phylum level, as well as sequences classified as eukaryotic, archaeal, mitochondrial or chloroplast, were discarded. Samples that retained fewer than 5,000 reads after taxonomical filtering were removed from further analyses (*n* = 15), leaving 39 samples in the study (20 HCs and 19 PD).

Phyloseq objects were generated using the phyloseq package v 1.46 (McMurdie and Holmes, 2013). Sequences classified as belonging to the same species or sequences unclassified at the species level were agglomerated to a consensus ASV.

#### 2.9.2 Bacterial composition and diversity analysis

Genus-level counts were converted to relative abundances for descriptive barplots [ggplot2 v 3.5.2 (Wickham, 2016) and fantaxtic v 0.2.1]. α-diversity indices (Chao1, ACE, Fisher, Shannon and Inverse Simpson) were calculated using vegan v 2.6–4 (Oksanen et al., 2022), while Faith’s phylogenetical distance (Faith’s PD) was computed using the picante package v 1.8.2 (Kembel et al., 2010), after rarefying reads to 5,171 reads/sample.

UniFrac distances were calculated from raw counts after prevalence filtering (10% of samples) and rarefaction using GUniFrac v 1.8 (Chen et al., 2012). Bray–Curtis distances were computed in phyloseq from the same abundance data. PERMANOVA was performed using vegan (999 permutations). The analysis was based on Aitchison distance matrices, calculated from centered log-ratio (CLR) transformed genus-level abundance data. The model included group status (HC vs. PD) as the primary variable of interest, along with covariates selected based on clinical relevance and potential confounding effects: age, sex, smoking status, glycemia, HDL, triglyceride levels, hepatic comorbidity, cardiovascular comorbidity, obesity, and lifestyle.

sPLS-DA were conducted using the package mixOmics v 6.26.0 (Rohart et al., 2017), from CLR-transformed genus-level abundance data. The final model was constructed with two components, and the number of selected features was set to 10 and 100 taxa for components 1 and 2, respectively, as determined through iterative model refinement. Model performance was evaluated using 5-fold M-fold cross-validation repeated 50 times, and classification accuracy was assessed across three distance metrics (maximum, centroid, and Mahalanobis). Feature loadings for each component were extracted to identify the most discriminative taxa contributing to group separation. Covariate-adjusted taxon-level estimates we assessed using linear models with empirical-Bayes moderation using the package limma v 3.58.1 m on CLR-transformed genus abundances. Limma models were run both without covariates, for direct comparison with exploratory methods, and with age and sex adjustment. For each taxon we calculated the estimated log fold-change (logFC, CLR units), the t statistic and the Benjamini–Hochberg (BH) false discovery rate (FDR) adjusted *p*-value.

### 2.10 Statistical analysis

Normality of continuous features was tested using the Shapiro–Wilk test. Normally distributed variables were compared with two-sample *t*-test; non-normally distributed variables were compared by Wilcoxon rank-sum test. All multiple comparisons used the BH FDR correction, with an FDR threshold of 0.05, unless otherwise stated. Categorical variables were compared using Fisher’s exact test when any cell count was < 5 or Pearson’s chi-squared test.

Differences in α-diversity indices and genus-level relative abundances were assessed by Wilcoxon rank-sum test with FDR correction. LEfSe, as implemented in the microbiomeMarker package v 1.8.0 (Cao et al., 2022), was used for assessing differentially abundant taxa between groups from genus-agglomerated phyloseq objects, using an LDA score cutoff of 4.1 and an FDR-adjusted Kruskal-Wallis *p*-value cutoff of 0.01.

Multicollinearity among sample features was assessed via variance inflation factors (VIFs), calculated using the car package v 3.1–3. Only variables with VIF < 3.5 were retained for model building. Post-hoc power calculations were performed using the pwr package v1.3–0.

## 3 Results

### 3.1 Anthropometric and metabolic parameters

Laboratory and clinical data revealed several notable differences between PD patients and HCs (Table 1). While no significant sex differences were observed between groups (χ^2^ = 2.07, *p* = 0.15), PD patients were on average older than HCs (mean age: 67.2 vs. 59.9 years), with this difference approaching statistical significance (*p* = 0.058). Fasting glycemia levels were significantly lower in the PD group compared to HCs (*p* = 0.02), as were total cholesterol (*p* = 0.027) and LDL-cholesterol concentrations (*p* = 0.047), suggesting that these parameters may act as potential confounders in downstream analyses of gut microbial composition. No significant group differences were detected for HDL-cholesterol (*p* = 0.119) or triglyceride levels (*p* = 0.877).

**Table 1:** Demographic, anthropometric, metabolic, and confounder information of the study participants.

		Entire group (*N* = 39)	HC (*N* = 20)	PD (*N* = 19)	*p*-value	Observations
Gender	M: F	19:20	7:13	12:7	0.15	Chi-squared test
Age (years)	M (SD)	63.46 (12.11)	59.9 (12.11)	67.21 (11.22)	0.058	*t*-test
	Range	37–89	37–76	48–89		
Glycemia (mg/dL)	M (SD)	98.47 (34.72)	104.8 (43.63)	91.82 (21.08)	**0.02**	Wilcoxon rank-sum test
	Range	48–272	48–272	71–157		
Cholesterol (mg/dL)	M (SD)	192.61 (50.39)	209.75 (48.05)	174.57 (47.47)	**0.027**	*t*-test
	Range	111–321	141–321	111–280		
HDL (mg/dL)	M (SD)	49.54 (16.12)	52.75 (15.79)	46.15.81 (16.19)	0.119	Wilcoxon rank-sum test
	Range	26.02–86.8	30–84	26.02–86.8		
LDL (mg/dL)	M (SD)	124.8 (47.37)	141.5 (50.24)	107.22 (37.9)	**0.047**	Wilcoxon rank-sum test
	Range	55.32–263	65–263	55.32–208.12		
Triglycerides (mg/dL)	M (SD)	134.3 (71.6)	138.85 (73.69)	129.5 (71.02)	0.877	Wilcoxon rank-sum test
	Range	57–373.51	57–339	60.99–373.51		
Smoking	Y: N	9:30	3:17	6:13	0.396	Chi-squared test
Diet					**<0.001**	Fisher’s Exact test
	Common	28	20	8		
	Low sodium	10	0	10		
	Hepatic	5	0	5		
	Other	7	0	7		
Lifestyle					**0.008**	Fisher’s Exact test
	Active	2	2	0		
	Minimal activity	5	5	0		
	Sedentary	32	13	19		
Comorbidities						
	Diabetes	5	0	5	**0.02**	Fisher’s Exact test
	Cardiovascular	10	0	10	**<0.001**	Fisher’s Exact test
	Hepatic	5	0	5	**0.02**	Fisher’s Exact test
	Obesity	5	2	3	0.661	Fisher’s Exact test

*p* values less than 0.05 given in bold.

Smoking prevalence did not differ significantly between groups (χ^2^ = 0.72, *p* = 0.396), although PD participants tended to report higher smoking rates (31.6%) than controls (15%). In terms of dietary habits, a significant divergence was noted (Fisher’s exact test, *p* < 0.001): all HCs followed a common (unrestricted) diet, whereas PD patients adhered to a variety of restrictive regimens including low sodium, hepatic, and hypoglycemic plans. These diets were largely prescribed in response to comorbid conditions and may contribute to differences in microbiota profiles. Lifestyle data further highlighted disparities between groups (Fisher’s exact test, *p* = 0.008). While all HCs reported either sedentary or minimally active routines, all PD participants reported a sedentary lifestyle, consistent with the mobility limitations often associated with the disease. Finally, comorbidity prevalence differed substantially across groups: cardiovascular disease (*p* < 0.001), hepatic disorders (*p* = 0.02), and diabetes mellitus (*p* = 0.02) were significantly more common in PD patients. Obesity, by contrast, was equally distributed between groups (*p* = 0.661).

Among the 19 PD patients included in the final analysis, there were differences observed in scale completion: HAM-A and HAM-D in PD group: [mean ± standard deviation (SD)] − 26.00, *n* = 1 compared with 30.66 ± 3.21, *n* = 3, indicating moderate to severe anxiety and depression. Scale scores on MMSE suggest that patients are experiencing moderate cognitive impairment, 15.22 ± 5.89, *n* = 9, moderate psychopathology as indicated on BPRS, 49.25 ± 6.99, *n* = 4 and moderately severe/very severe cognitive decline based on the Reisberg scale 6.33 ± 1.15, *n* = 3, respectively, significantly impaired capacity for self-determination/service according to GAFS 21.00 ± 10.13, *n* = 4. The main categories of controlled pharmacological compounds that the patients were prescribed included benzodiazepines, antidepressants, antipsychotics, nootropics, anti-epileptics/mood stabilizers, hypnotics, cognitive enhancers, and anticholinergics. Given the diversity of treatment strategies, we chose to centralize the drugs more generally.

### 3.2 Bacterial composition

**Figure 1:**
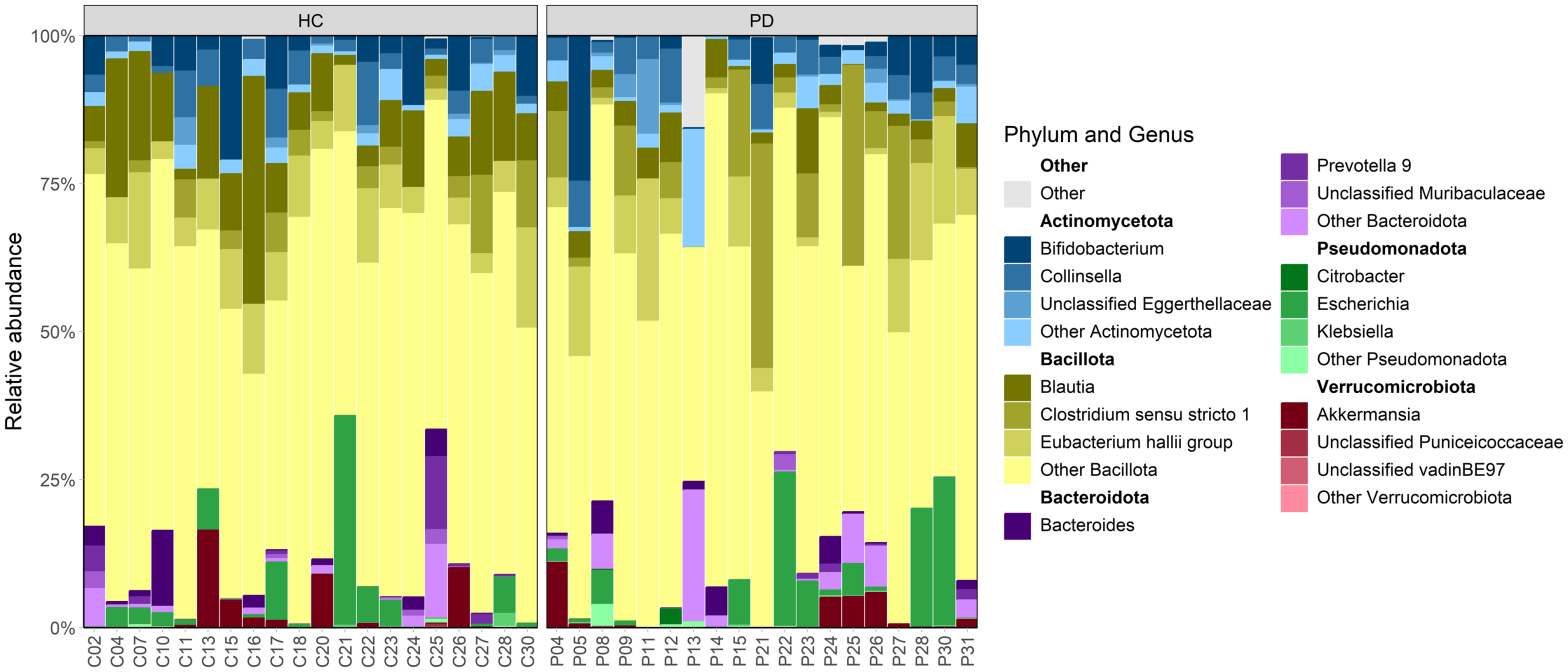
Comparative overview of dominant phylum and genus-level microbial composition in PD and HC individuals; corresponding phyla highlighted in bold above each genus group.

The successive stages of quality filtering sequences have reduced the number of participants to 39, resulting in a study group comprising 19 PD and 20 HC subjects. Across these samples, a total of 8,855,982 raw sequences were read, with an average of 227,076 and a median of 107,210 reads per sample. Following quality control and filtering steps (e.g., removal of chimeras, low-quality reads and non-bacterial sequences), the number of reads was reduced to 3,772,510 (57.4% sequences were discarded). The final feature table contained 498 unique bacterial ASVs across all 39 samples. Figure 1 illustrates the abundance of the three most common bacterial genera from the five most abundant phyla in each analyzed sample.

Both groups showed a higher prevalence of bacteria from the phyla Bacillota and Actinomycetota, while exhibiting lower relative abundances of Bacteroidota, Pseudomonadota, and Verrucomicrobiota members. In terms of genus-level relative abundances, no statistically significant differences were observed between PD and HC groups. However, the family *Lachnospiraceae*, part of the Bacillota phylum, was found to be more abundant in HCs (36.13 ± 2.71%) compared to PD (20.74 ± 2.33%), with an FDR-adjusted *p*-value of 0.0156. Genera within the phylum Actinomycetota, particularly *Bifidobacterium* and *Collinsella*, were present at moderate to low abundance across most samples, as was the genus *Akkermansia* from the phylum Verrucomicrobiota. Although not significantly different in our study in terms of relative abundance, these bacterial genera have previously been reported to be differentially abundant when comparing PD versus HCs (Li Z. et al., 2023).

### 3.3 Bacterial diversity

**Figure 2:**
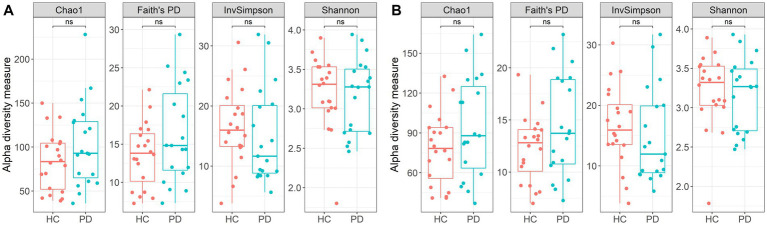
Alpha diversity metrics: Faith’s phylogenetic diversity, inverse Simpson index, observed number of unique sequences and Shannon index on **(A)** all sequences; **(B)** after rarefaction; ns – not significant (FDR-adjusted *p*, Wilcoxon rank-sum test).

The two study groups did not differ significantly across calculated α-diversity metrics, including phylogenetic diversity, as measured by Faith’s PD (Figure 2A). Given that library size can influence α-diversity estimates (Willis, 2019), read counts were also rarefied to the minimum number observed across all samples (5,171 reads). Even after rarefaction, group-level differences remained non-significant (Figure 2B). It is important to note that while Chao1 and ACE indices are frequently used as richness indicators, their application in ASV datasets has been questioned (Deng et al., 2024), as they may underestimate true richness and therefore be interpreted cautiously in this context.

**Figure 3:**
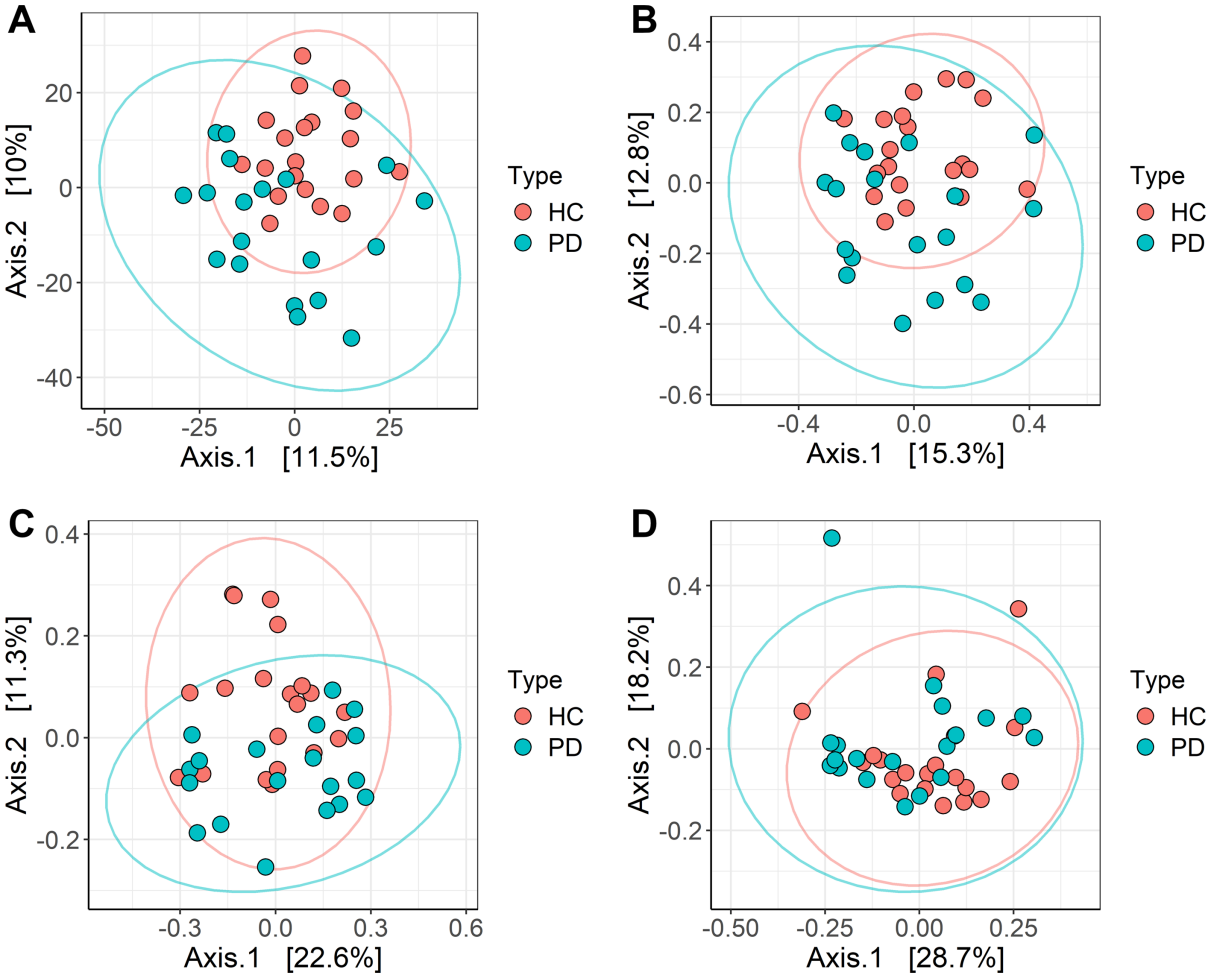
PCoA analysis based on **(A)** Aitchinson distance, **(B)** Bray-Curtis distance, **(C)** unweighted Unifrac distances, and **(D)** weighted Unifrac distances. Colors represent sample types: red – HC; blue – PD; ellipses are drawn at 95% confidence level. The percentage of variation explained by the first two dimensions is indicated on respective axes.

Among the four examined β-diversity metrics, Aitchison distance (Figure 3A) revealed the most pronounced separation between groups after PCoA. Bray–Curtis (Figure 3B) and unweighted UniFrac (Figure 3C) showed only modest separation, while weighted UniFrac led to substantial overlap between samples (Figure 3D). The lack of separation based on weighted UniFrac is consistent with the non-significant Faith’s PD results, as both metrics incorporate phylogenetic relationships and are more sensitive to shifts in abundant taxa. Conversely, the clearer group separation with Aitchison distance suggests that compositional changes rather than shifts in phylogenetic structure are more characteristic of the microbial differences associated with PD. These findings highlight the importance of selecting distance metrics that are appropriate to the data structure and study aims.

**Figure 4:**
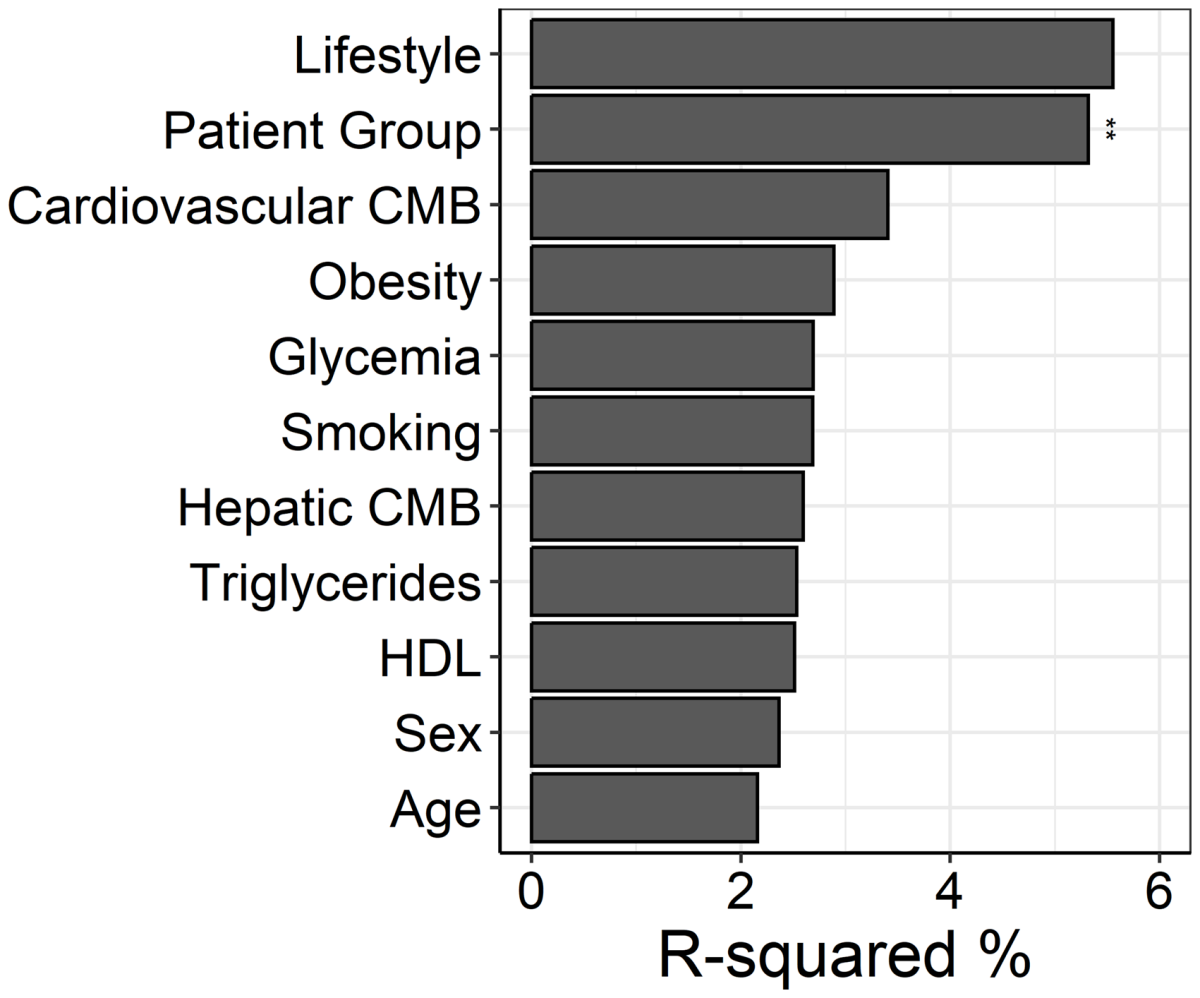
Adonis PERMANOVA comparison with metadata for entire study population; R-squared refers to amount of Aitchinson variation associated with each category; **p < 0.01.

We next assessed community-level differences between groups with PERMANOVA based on the Aitchinson distance (Figure 4). The analysis revealed a significant effect of disease status (*R*^2^ = 5.3%, *p* = 0.002), even after correcting for clinical covariates such as age, sex, biochemical parameters and comorbidities. Age explained 2.16% of variance (*p* = 0.753), and sex explained 2.36% (*p* = 0.625). While none of the individual covariates significantly explained variation in microbial composition, cardiovascular comorbidity showed a borderline association (*p* = 0.091).

**Figure 5:**
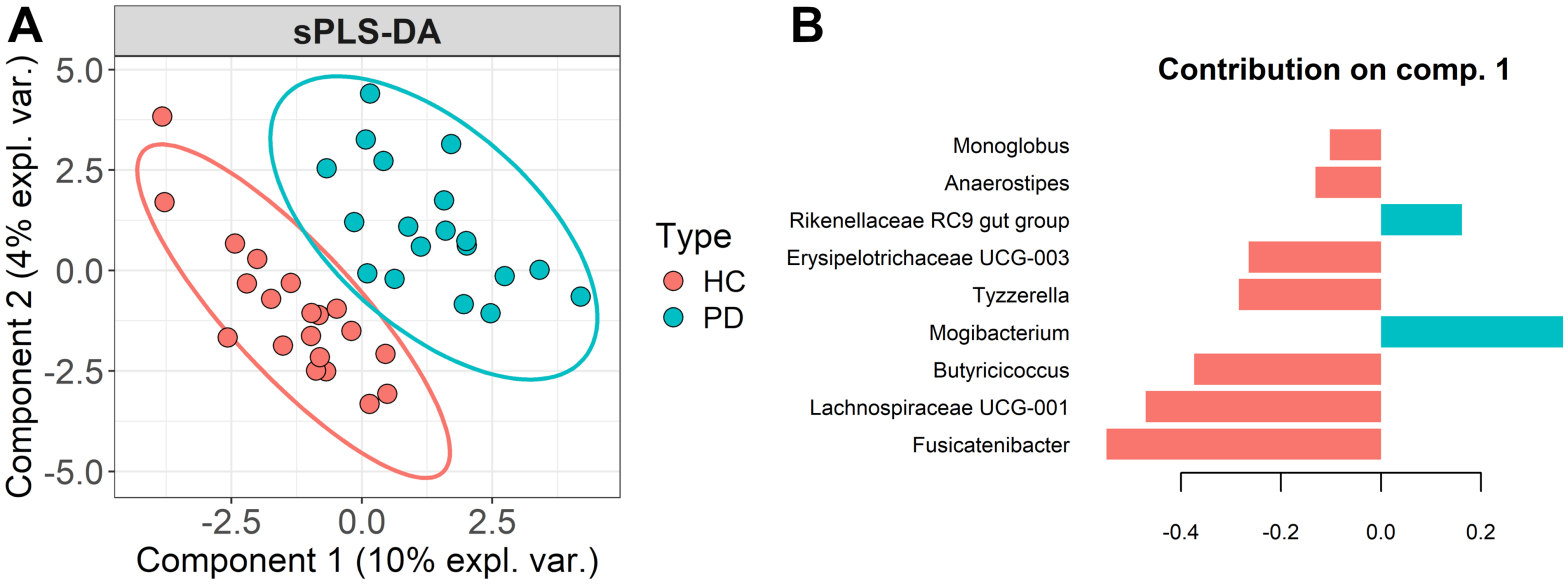
**(A)** sPLS-DA bi-plot. **(B)** Top 9 most relevant contributions for PC1 separation.

To further investigate group-level discriminative taxa, sPLS-DA was applied to CLR-transformed genus-level abundance data. The model showed a good separation between groups (Figure 5A), with an overall classification error rate of 26.8% using two components (class-specific error HC 20.4%, PD 34.2%), indicating a more accurate classification of HC samples and suggesting greater inter-individual heterogeneity in the PD group. Separation of PD patients on Component 1 was primarily driven by the genera *Mogibacterium* and *Rikenellaceae RC9 gut group* (Figure 5B). In contrast, the discriminant genera contributing to HC group classification included several SCFA-producing genera such as *Fusicatenibacter, Lachnospiraceae UCG-001, Butyricicoccus*, *Anaerostipes,* as well as *Erysipelotrichaceae UCG-003,* and *Tyzzerella,* taxa which have been implicated in gut homeostasis and anti-inflammatory activity. Notably, *Fusicatenibacter* and other members of the *Lachnospiraceae* family have previously been shown to be less abundant in PD patients, while members of the *Rikenellaceae* family have been associated with the PD phenotype (Li Z. et al., 2023). For the smallest observed effect among the top three genera contributing to Component 1 (Cohen’s *d* = 1.11), we estimated 14 subjects per group for 80% power (*α* = 0.05). With our actual cohort sizes (*n* = 19 and *n* = 20), the achieved power for detecting *d* = 1.11 is approximately 92% (two-sided *t*-test, *α* = 0.05), indicating that our sample is sufficiently powered for effects of this magnitude.

**Figure 6:**
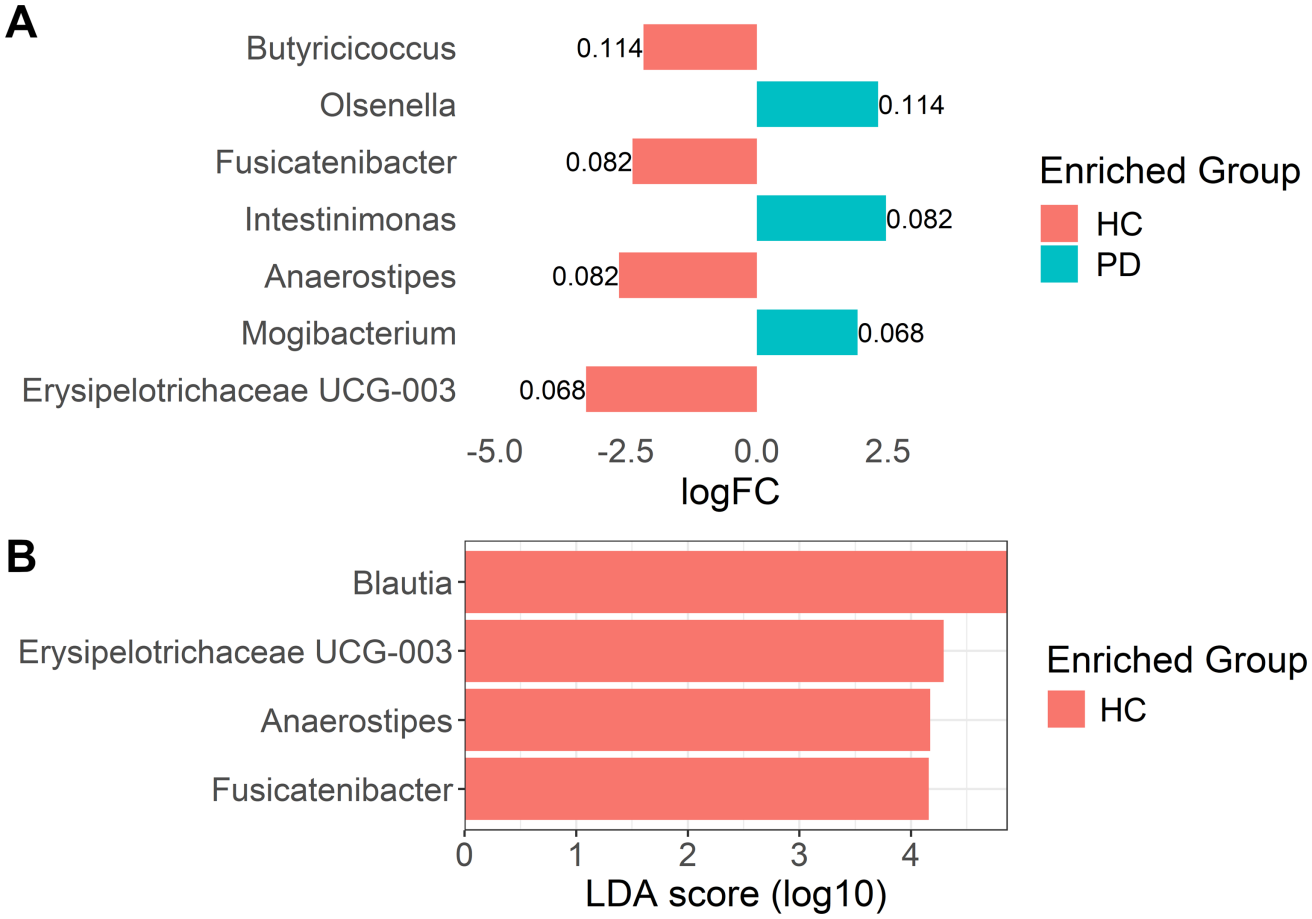
**(A)** Limma-estimated covariate-adjusted log fold-changes for top taxa; labels are FDR-adjusted *p*-values. **(B)** Results of differential abundance analysis by LEfSe.

We complemented the sPLS-DA results using linear models on CLR-transformed abundances. In models including only patient group, the genera identified among the top hits were identical to the sPLS-DA contributions in Component 1, with FDR-adjusted *p* ~ 0.042. However, when analyses were adjusted for age and sex, the ranking of top taxa changed slightly: *Erysipelotrichaceae UCG-003* and *Mogibacterium* rose to the top (Figure 6A), and although these taxa retained large effect sizes, their FDR-adjusted *p*-values were attenuated (0.068–0.082) and therefore did not meet a strict FDR-adjusted *p* < 0.05 threshold after covariate adjustment. This indicates that some unadjusted associations are partly explained by age or sex. Nevertheless, several genera remain among the strongest candidates for group differences even after adjustment.

LEfSe analysis confirmed the enrichment of several HC-associated taxa, identifying four genera with significantly higher abundance in the HC group (LDA score > 4.1, adjusted *p* < 0.01) (Figure 6B). No genera were found to be significantly more abundant in the PD group, again emphasizing group heterogeneity. Importantly, the LEfSe-identified taxa overlapped with those found via sPLS-DA and linear models, and additionally *Blautia*, another known SCFA-producing genus.

Together, our findings underscore a consistent depletion of key SCFA-producing genera in PD patients, and highlight the value of combining multivariate and univariate methods for robust detection of both global patterns and taxon-specific differences in complex datasets.

## 4 Discussion

This pilot study, conducted at a tertiary care center, sequenced the V3–V4 region of the 16S rRNA gene from stool samples of patients with PD and HCs. The analysis revealed distinct and overlapping microbial alterations at the phylum level. Both groups exhibited relatively high abundances of Bacillota and Actinobacteriota (Li et al., 2017; Zapała et al., 2021; Nuzum et al., 2023), phyla involved in maintaining mucosal integrity, SCFAs production, and immune modulation (Li et al., 2017; Zapała et al., 2021; Nuzum et al., 2023; Zhang et al., 2023). Nevertheless, PD patients consistently showed a reduced relative abundance of Bacteroidota (Bedarf et al., 2017), a phylum associated with anti-inflammatory signaling and preservation of the intestinal barrier (Li et al., 2017; Zhang et al., 2020; Yan et al., 2021).

Conversely, an enrichment of Proteobacteria, comprising endotoxin-producing Gram-negative bacteria implicated in gut inflammation, has been observed in PD cohorts (Weis et al., 2019; Melis et al., 2021; Yan et al., 2021). Notably, several studies report increased levels of Verrucomicrobiota, particularly *Akkermansia muciniphila*, in PD (Bedarf et al., 2017). The overrepresentation of this mucin-degrading taxon may reflect a compensatory response to altered mucus production or dysregulated mucosal signaling (Barichella et al., 2019; Sarah et al., 2020; Zhang et al., 2020; Yan et al., 2021; Nuzum et al., 2023), although emerging evidence also suggests that excessive *Akkermansia* may compromise barrier integrity, potentially exacerbating neuroinflammation (Keshavarzian et al., 2015; Forsyth et al., 2011; Patel and Pyrsopoulos, 2019).

Despite inter-individual variability influenced by geographic, dietary, and environmental factors, studies employing age- and spouse-matched controls have demonstrated reproducible microbial signatures, suggesting that these differences are not solely due to lifestyle factors (Zhang et al., 2020, 2023; Kenna et al., 2021). However, demographic heterogeneity across cohorts underscores the need for geodemographically stratified microbiome research (Zhang et al., 2020, 2023; Babacan Yildiz et al., 2023).

Conventional dopaminergic strategies, including levodopa and entacapone, can modulate gut microbial composition, potentially confounding microbiota analyses (Weis et al., 2019; Melis et al., 2021; Fu et al., 2022a). Melis et al. (2021) showed that both oral levodopa and levodopa-carbidopa intestinal gel increased Proteobacteria while reducing Bacillota, while Fu et al. (2022a) demonstrated that entacapone altered gut microbial structure and impacted amino acid metabolic pathways. Nonetheless, Bedarf et al. (2017) identified microbiota changes in L-DOPA-naïve PD patients, indicating that dysbiosis may arise independently of medication. This is further supported by additional studies reporting alterations in treatment-naïve, *de novo* PD patients, implying that microbial shifts may precede clinical onset (Bedarf et al., 2017; Boertien et al., 2022).

As the role of the GBA axis in PD becomes clearer, several microbiome-targeted interventions have gained attention. Fecal microbiota transplantation (FMT) has shown potential to improve motor and non-motor symptoms in small-scale trials (Kuai et al., 2021; DuPont et al., 2023). Similarly, adherence to the Mediterranean diet is associated with improved GI function and favorable microbial modulation (Rusch et al., 2021), while adjunct probiotic supplementation has been linked to symptom relief and anti-inflammatory effects (Ghyselinck et al., 2021; Sun H. et al., 2022; Gorecka-Mazur et al., 2024).

Among Bacillota, the genus *Blautia* was detected in all samples but was significantly more abundant in HCs (Keshavarzian et al., 2015; Li et al., 2017; Aho et al., 2019; Wallen et al., 2020; Ren et al., 2020; Sarah et al., 2020; Ji et al., 2021; Melis et al., 2021; Guo et al., 2022; Nishiwaki et al., 2022; Pavan et al., 2023). Other genera such as *Fusicatenibacter* and *Erysipelotrichaceae UCG-003* also differed between groups but remained low in relative abundance (Weis et al., 2019; Wallen et al., 2020; Lubomski et al., 2022a; Lubomski et al., 2022b; Nishiwaki et al., 2022; Babacan Yildiz et al., 2023; Pavan et al., 2023). These taxa, particularly *Blautia*, are recognized SCFA producers, mainly butyrate (Keshavarzian et al., 2015), which is essential for maintaining intestinal epithelial integrity (Pavan et al., 2022).

The reduction of SCFA-producing genera has been linked to both motor and non-motor PD symptoms, including mild cognitive impairment (MCI) (Ren et al., 2020). These bacteria may also exert inflammatory effects (Chen et al., 2021; Zhang et al., 2021; Fu et al., 2022b), constipation, and serve as potential biomarkers of disease progression (Nishiwaki et al., 2022). They contribute to fermentation of resistant starches and degradation of dietary fibers like cellulose (Takahashi et al., 2016), and their abundance often increases following FMT (Kuai et al., 2021; DuPont et al., 2023). For instance, *Lachnospiraceae UCG-001* is a butyrate producer with known anti-inflammatory properties (Almamoun et al., 2023), tends to increase with age (Kim et al., 2021), and may respond positively to probiotic supplementation (Gryaznova et al., 2022), with possible relevance for metabolic conditions such as type 2 diabetes (Wei et al., 2018).

The genus *Collinsella*, part of the core fecal gut microbiota, has been reported to increase in individuals experiencing weight loss, including PD patients (Del Chierico et al., 2020), while being less abundant in obese individuals with type 2 diabetes, highlighting its metabolic sensitivity. Diet appears to modulate its abundance: it is promoted by high-protein, low-fat and low-carbohydrate diets (Clavel et al., 2014), and rises with low fiber intake (Gomez-Arango et al., 2018). Although generally low in abundance in both PD and HCs groups, similar in an Indian cohort (Pavan et al., 2023), multiple studies report increased *Collinsella* in PD patients (Cirstea et al., 2020; Zhang et al., 2020; Chen et al., 2021; Li et al., 2021), including post-FMT (Kuai et al., 2021), suggesting that its elevation may reflect disease-associated gut changes rather than dietary influences alone.

Together with *Collinsella* and *Akkermansia* (Dumitrescu et al., 2021; Lee et al., 2021), *Bifidobacterium* has emerged as a genus of interest due to its role in carbohydrate metabolism and anti-inflammatory functions, which are particularly relevant in PD given the frequent compromise of gut barrier integrity (Dumitrescu et al., 2021). Reduced levels of *Bifidobacterium* have been associated with worsened PD symptoms (Minato et al., 2017), and supplementation has been shown to improve microbial balance and relieve GI symptoms, especially constipation (Barichella et al., 2016; Baldini et al., 2020). Deficiency in this genus has also been linked to increased inflammation (Li et al., 2021), cognitive decline (Cilia et al., 2021), and altered SCFA metabolism, notably an elevated plasma-to-cell butyrate ratio observed in dementia (Chen et al., 2022).

However, the contribution of *Bifidobacterium* to chronic constipation remains unclear, with some studies yielding conflicting results (Rajilić-Stojanović et al., 2011; Chassard et al., 2012). Its role in PD is similarly complex. While several studies report increased abundance of *Bifidobacterium* in PD (Petrov et al., 2017; Lin et al., 2018; Aho et al., 2019, 2021; Barichella et al., 2019; Wallen et al., 2020; Cirstea et al., 2020; Sarah et al., 2020; Yan et al., 2021; Ji et al., 2021; Jo et al., 2022; Nishiwaki et al., 2022; Shi et al., 2023), interpretations vary: this increase may represent a compensatory response to dysbiosis or be medication-induced. Conversely, other studies report either no significant change (Aho et al., 2019; Pereira et al., 2022; Nuzum et al., 2023), or a decrease in abundance (Li F. et al., 2019; Del Chierico et al., 2020; Ngo et al., 2020; Boertien et al., 2022; Mehanna et al., 2023). These inconsistencies highlight the influence of confounding variables, including dopaminergic treatment (Unger et al., 2016; Hill-Burns et al., 2017; Weis et al., 2019; Hughes et al., 2020; Wallen et al., 2020; Lubomski et al., 2022a), sex-specific microbiome differences (Gao et al., 2018), and geographic diversity in microbial composition (Hasegawa et al., 2015; Keshavarzian et al., 2015).

*Akkermansia*, despite its known role in maintaining host homeostasis (Kong et al., 2016), shows a nuanced pattern in PD. Many studies report increased levels of PD (Keshavarzian et al., 2015; Unger et al., 2016; Bedarf et al., 2017; Heintz-Buschart et al., 2018; Li C. et al., 2019; Li F. et al., 2019; Lin et al., 2019; Baldini et al., 2020; Nishiwaki et al., 2020a, 2022; Sarah et al., 2020; Wallen et al., 2020; Zhang et al., 2020, 2022; Cirstea et al., 2020; Zapała et al., 2021; Kenna et al., 2021; Babacan Yildiz et al., 2023). It contributes to GI function and immune regulation primarily through mucin degradation (Reunanen et al., 2015; Ottman et al., 2017; Plovier et al., 2017; Geerlings et al., 2018), which occurs in the outer mucus layer adjacent to intestinal epithelial cells (Hertel et al., 2019; Baldini et al., 2020). While its metabolic byproducts may supply energy to commensals, they can also promote inflammation when *Akkermansia* levels rise while SCFA concentrations drop (Forsyth et al., 2011; Aho et al., 2021). Conversely, excessive *Akkermansia* may compromise barrier integrity (Patel and Pyrsopoulos, 2019), potentially facilitating α-syn accumulation within the ENS (Keshavarzian et al., 2015; Fujio-Vejar et al., 2017). This imbalance has been linked to various non-motor PD symptoms, including slowed GI transit, firmer stools, and increased constipation severity (Gobert et al., 2016; Vandeputte et al., 2016; Cirstea et al., 2020; Pavan et al., 2022, 2023).

Finally, it is important to note that while traditional diversity metrics failed to reveal statistically significant differences between PD and HC groups, sPLS-DA and LEfSE successfully identified distinct microbial signatures that differentiate the two cohorts. Neither α-diversity (including phylogenetic diversity, richness, or evenness indices) nor global β-diversity metrics such as Bray–Curtis or UniFrac distances demonstrated consistent or robust group separation. This suggests that overall community diversity and phylogenetic structure may not differ markedly between groups at the global level. In contrast, sPLS-DA, which emphasizes feature selection and supervised discrimination, was able to uncover subtle but biologically meaningful compositional shifts by identifying specific genera that contribute to between-group variance. These findings illustrate a key advantage of supervised, log-ratio-aware multivariate methods in detecting targeted changes that are not captured by unsupervised diversity metrics. The results support the notion that PD-associated dysbiosis may not manifest through gross alterations in diversity but rather through nuanced shifts in the relative abundance of key microbial taxa, particularly those involved in SCFA production and mucosal health.

### 4.1 Limitations and highlights of the study

This study has several limitations that should be considered when interpreting the findings. First, the relatively small sample size may limit statistical power, particularly for diversity-based metrics. Second, the cohort was geographically and demographically homogeneous, which may reduce generalizability to broader PD populations. Third, while sPLS-DA identified taxa with strong discriminative power, the method is inherently sensitive to data preprocessing and parameter tuning; thus, results should be interpreted in the context of CLR transformation and the specific model configuration used. Additionally, the absence of significant α- or β-diversity differences highlights that global community shifts may be subtle or masked by high inter-individual variability. Notably, although sex and age were accounted for in the multivariate PERMANOVA model, the inverse female-to-male ratio between PD and HC groups can be regarded as a limitation of the study, given their known influence on gut microbial composition. Our reported power estimates are per-taxon (unadjusted); multiple-testing correction reduces effective discovery power and therefore larger cohorts are required to detect medium and small effects. Finally, the cross-sectional design limits causal inference, and future longitudinal studies with larger, multi-site cohorts are needed to validate the microbial markers identified and explore their role in PD pathophysiology over time.

## 5 Conclusion

This pilot study identified distinct compositional features in the gut microbiota of PD patients compared to HCs, despite considerable inter-individual and environmental variability. Both groups shared a core microbiome dominated by Bacillota and Actinomycetota, but HC samples consistently exhibited higher relative abundances of SCFA-producing genera such as *Blautia*, *Fusicatenibacter*, and *Erysipelotrichaceae UCG-003*. These taxa have been implicated in intestinal mucosal integrity and anti-inflammatory activity, suggesting a potential protective role.

PD samples were characterized by reduced presence of these beneficial bacteria and a relative enrichment of *Rikenellaceae RC9* gut group and *Mogibacterium*, taxa previously linked to PD-associated dysbiosis. The potential involvement of *Rikenellaceae* in PD pathophysiology remains unclear and merits further investigation, particularly given the conflicting findings in the current literature.

While α-diversity did not differ significantly between groups, β-diversity analyses, particularly Aitchison distance revealed significant compositional divergence, indicating that microbiome alterations in PD are more reflective of community structure than phylogenetic diversity. Multivariate and univariate analyses yielded converging evidence for disease-specific microbial signatures, underscoring the relevance of *Lachnospiraceae* taxa as discriminators of PD status. Importantly, no genera were significantly enriched in PD patients, highlighting both the heterogeneity and potential depletion of functionally important bacteria in this group.

These findings reinforce the emerging hypothesis that reduced SCFA production may contribute to the pathophysiology of PD and support the utility of microbiome-targeted approaches as adjunctive therapeutic strategies. Further large-scale, geodemographically controlled studies are warranted to validate these associations and elucidate causal relationships.

## Data Availability

The datasets presented in this study can be found in online repositories. The names of the repository/repositories and accession number(s) can be found at: https://zenodo.org/records/15647546. The scripts used for the generation of presented results and figures are available upon request from corresponding author.
